# A Decade of Croup Management: Guideline Adherence, Practice Variation, and Opportunities for Improvement

**DOI:** 10.3390/children13081046

**Published:** 2026-08-06

**Authors:** Jirattikarn Jirawong, Wanaporn Anuntasaree, Kanokpan Ruangnapa, Kantara Saelim, Pharsai Prasertsan

**Affiliations:** 1 Department of Pediatrics, Faculty of Medicine, Prince of Songkla University, Songkhla 90110, Thailand; jirattikarn.jir@gmail.com; 2 Division of Pediatrics Pulmonology and Critical Care Medicine, Department of Pediatrics, Faculty of Medicine, Prince of Songkla University, Songkhla 90110, Thailand; awanapor@medicine.psu.ac.th (W.A.); kanokpan.r@psu.ac.th (K.R.); kantara.s@psu.ac.th (K.S.)

**Keywords:** adherence, children, clinical practice guideline, croup, return visit

## Abstract

**Highlights:**

**What are the main findings?**

• Although there was a marked improvement in adherence to the national CPG for croup over the past decade, corticosteroid underuse and inappropriate ancillary treatments remain common.
• Incomplete adherence was associated with mild croup, outpatient visits, care by paediatricians, and earlier study years, whereas unplanned revisits were associated with care provided by general practitioners, emergency-department visits, afternoon presentations, and receipt of repeated epinephrine nebulisation in the first visit.

**What are the implications of the main findings?**

• There is a need for targeted educational and system-level interventions to improve adherence to the CPG, particularly for patients with mild croup managed in outpatient settings.
• National-level data would provide a clearer understanding of adherence to croup management among different settings and may help inform future guideline recommendations.

**Abstract:**

**Introduction**: Despite the availability of the national clinical practice guideline (CPG) for croup treatment, adherence has not been evaluated in real-world settings. This study aimed to evaluate adherence to the national CPG for croup and to identify factors associated with incomplete adherence. **Method**: Patients aged 1 month to 15 years diagnosed with viral croup between 1 January 2014 and 31 December 2023 were retrospectively reviewed. Complete adherence was defined as follows: (1) corticosteroid prescription for any severity, (2) nebulised epinephrine for moderate-to-severe cases, and (3) observation for ≥4 h for moderate-to-severe croup. Furthermore, associated factors for incomplete adherence and unplanned revisits within 72 h were explored. **Results**: A total of 437 visits were included; 71.2% were male patients, with a mean age of 25.6 ± 19 months. Complete adherence increased from 46.7% in 2014 to 87.6% in 2023. Corticosteroid prescription showed the lowest adherence rates, particularly in mild cases. Factors associated with incomplete adherence by univariable analyses included the first croup episode (odds ratio [OR], 2.37; 95% confidence interval [CI], 1.15–5.54), a paediatrician as the primary physician (OR, 2.61; 95% CI, 1.64–4.14), outpatient visits (OR, 2.36; 95% CI, 1.49–3.73), mild croup (OR, 1.89; 95% CI, 1.21–2.98), and earlier years of the study (OR, 2.41; 95% CI, 1.55–3.78). The unplanned revisit rate was 19.7% and was not associated with adherence. **Conclusions**: Adherence to the national guidelines increased over the past decade. However, suboptimal treatment practices underscore the need for targeted interventions to improve guideline-based care, particularly in paediatric and outpatient settings.

## 1. Introduction

Croup is a common respiratory infection in young children caused by respiratory viral pathogens, particularly parainfluenza virus type 1, human rhinovirus, and respiratory syncytial virus [1,2]. The cornerstone of viral croup management includes corticosteroids, nebulised epinephrine, and oxygen therapy. Corticosteroids significantly decrease the symptoms of upper airway obstruction within 2–6 h of administration. They have been shown to reduce hospital admissions, shorten the length of stay, and decrease the need for intubation compared with a placebo [3,4,5]. Thus, many national clinical practice guidelines (CPGs) recommend corticosteroid therapy for all patients diagnosed with croup [6].

In moderate-to-severe cases, nebulised epinephrine is another adjunctive treatment. It alleviates symptoms by causing vasoconstriction of the airway mucosa, thereby reducing mucosal oedema within 10–30 min [7]. In emergency departments (EDs), nebulised epinephrine has been associated with reduced hospital admissions compared with placebo [3,7,8,9]. However, the indications for its use differ among CPGs; some recommend it should be reserved for severe or life-threatening cases, while others allow its use for any patient with stridor, irrespective of severity [6].

**Figure 1:**
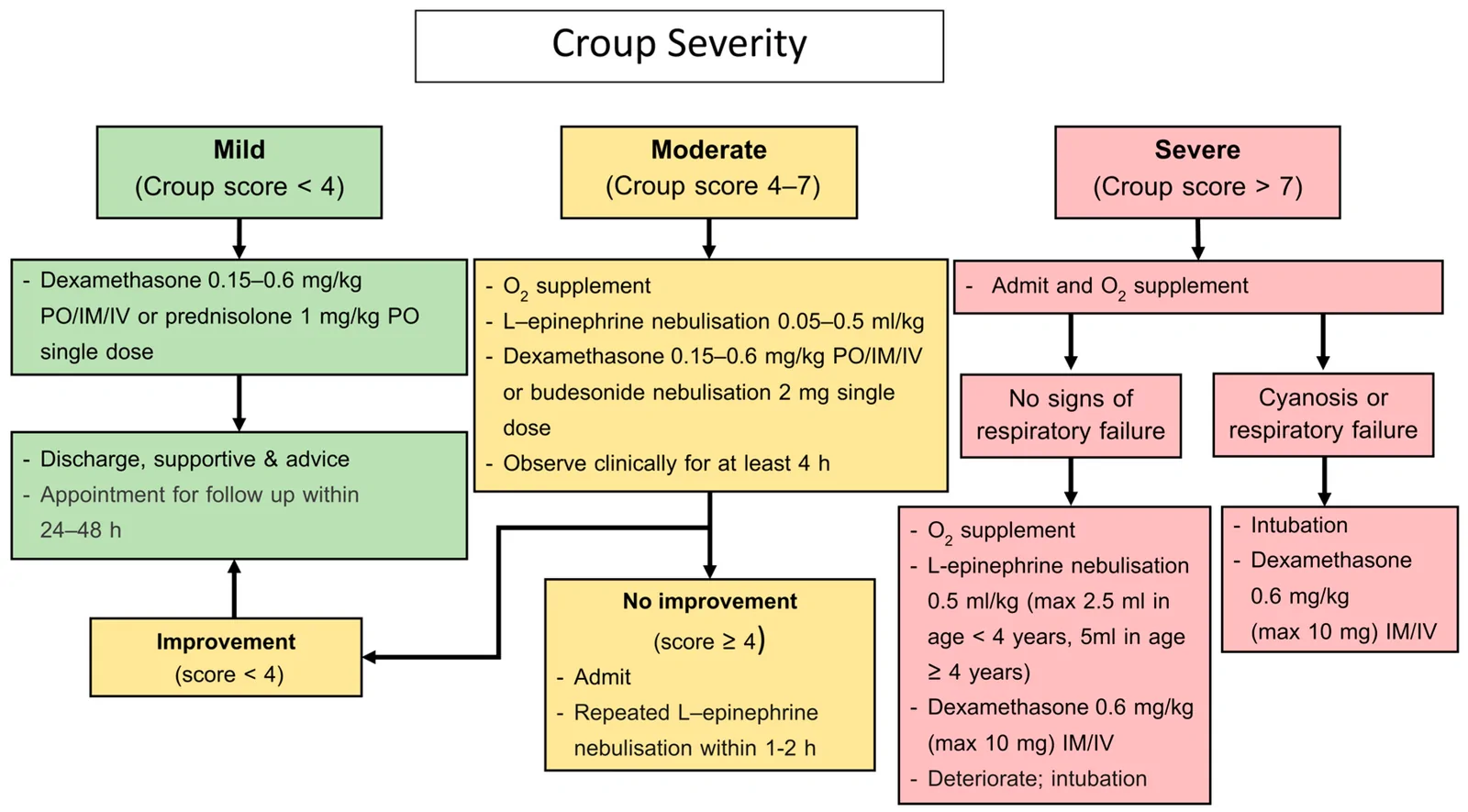
Thailand clinical practice guidelines for croup management, 2019.

In Thailand, the CPG for croup management has been developed by the Paediatric Respiratory and Critical Care Association of Thailand since 1996 and is updated every 5 years, with the most recent version published in 2019 [10]. The guideline categorises croup severity using the Downes Croup Score with mild (0–3 points), moderate (4–7 points), and severe (>7 points) levels. Each category has specific protocols for L-epinephrine nebulisation and corticosteroid treatment, as illustrated in Figure 1.

Despite the longstanding availability of CPGs for croup management in Thailand, no studies have evaluated adherence to these CPGs in real-world clinical settings. Therefore, this study aimed to assess adherence to the national CPGs for croup and to evaluate the risk factors associated with incomplete adherence and unplanned revisits.

## 2. Methods

### 2.1. Study Design, Setting, and Participants

This retrospective cohort study was conducted at a university-affiliated tertiary hospital in southern Thailand. Paediatric patients aged 1 month to 15 years who were diagnosed with croup-related conditions as defined by the International Classification of Diseases, 10th Revision, Clinical Modification (ICD-10-CM) codes J05.0 and J04.2 were included. Patients were excluded if they were referred from an outside hospital, had a history of congenital airway malformation, or had undergone laryngeal or tracheal surgery (Table S1). Study size was calculated according to the proportion of corticosteroid adherence reported in a previous study [11]; a minimum of 290 croup episodes were required for this analysis.

### 2.2. Ethics Statement

Ethical approval for this study was obtained from the Ethics Committee of the Faculty of Medicine, Prince of Songkla University, Songkhla, Thailand (REC.65-248-1-1). The requirement for informed consent was waived in view of the retrospective nature of the study, and all procedures performed were part of routine care. The report was written in accordance with the Strengthening the Reporting of Observational Studies in Epidemiology (STROBE) statement.

### 2.3. Data Collection and Measurements

After eligible patients were identified, manual chart reviews were conducted for all participants to exclude follow-up visits, misclassified croup diagnoses, and visits that satisfied the exclusion criteria. Baseline characteristics, including age, sex, history of preterm birth (defined as gestational age < 37 weeks), previous croup diagnosis, intubation history, and comorbidities, were extracted from the hospital information system (HIS). Information on croup diagnosis and management was also collected, including the initial croup severity score, location of the first visit (e.g., outpatient department [OPD] or ED), type of attending physician, type and dosage of corticosteroids, nebulised epinephrine, unnecessary intervention, and treatment outcomes. Corticosteroid and epinephrine doses were standardised to weight-based units. Corticosteroid overdose was defined as dexamethasone >0.6 mg/kg or >10 mg/dose or prednisolone >60 mg/dose, while underdosing was defined as dexamethasone <0.15 mg/kg/dose or prednisolone <1 mg/kg/dose. Epinephrine nebulisation (NB) overdose was defined as >2.5 mL/dose in children <4 years or >5 mL/dose in those ≥4 years. An underdose of epinephrine NB was defined as <0.05 mL/kg/dose. The observation time was calculated from the time of arrival to discharge. During the first visit, the observation time was recorded as ≥4 h if a patient was admitted. Unplanned revisits were defined as any follow-up hospital visit occurring within 72 h of initial discharge that required croup-related interventions or treatments.

The primary outcome was adherence to the national CPGs for croup management, which included (1) prescription of corticosteroids for any severity, (2) prescription of nebulised epinephrine for moderate-to-severe croup, and (3) observation time of ≥4 h for moderate-to-severe croup. Patients who met all three criteria were classified as having complete adherence. Second, we aimed to identify risk factors for incomplete adherence and unplanned revisits.

### 2.4. Statistical Analysis

Descriptive statistics are presented as percentages for categorical variables and as means with standard deviations (±SDs) or medians with interquartile ranges (IQRs), depending on the distribution of continuous data. Group comparisons used chi-square or Fisher’s exact tests for categorical variables and independent *t*-tests for continuous variables. Univariable logistic regression analyses were performed to assess the association between each factor and the outcome. Odds ratios (ORs) with 95% confidence intervals (CIs) were estimated to quantify the strength of the associations. Statistical significance was set at *p* < 0.05. All analyses were performed using R software, version 4.3.1 (The R Foundation for Statistical Computing, Vienna, Austria).

## 3. Results

**Figure 2:**
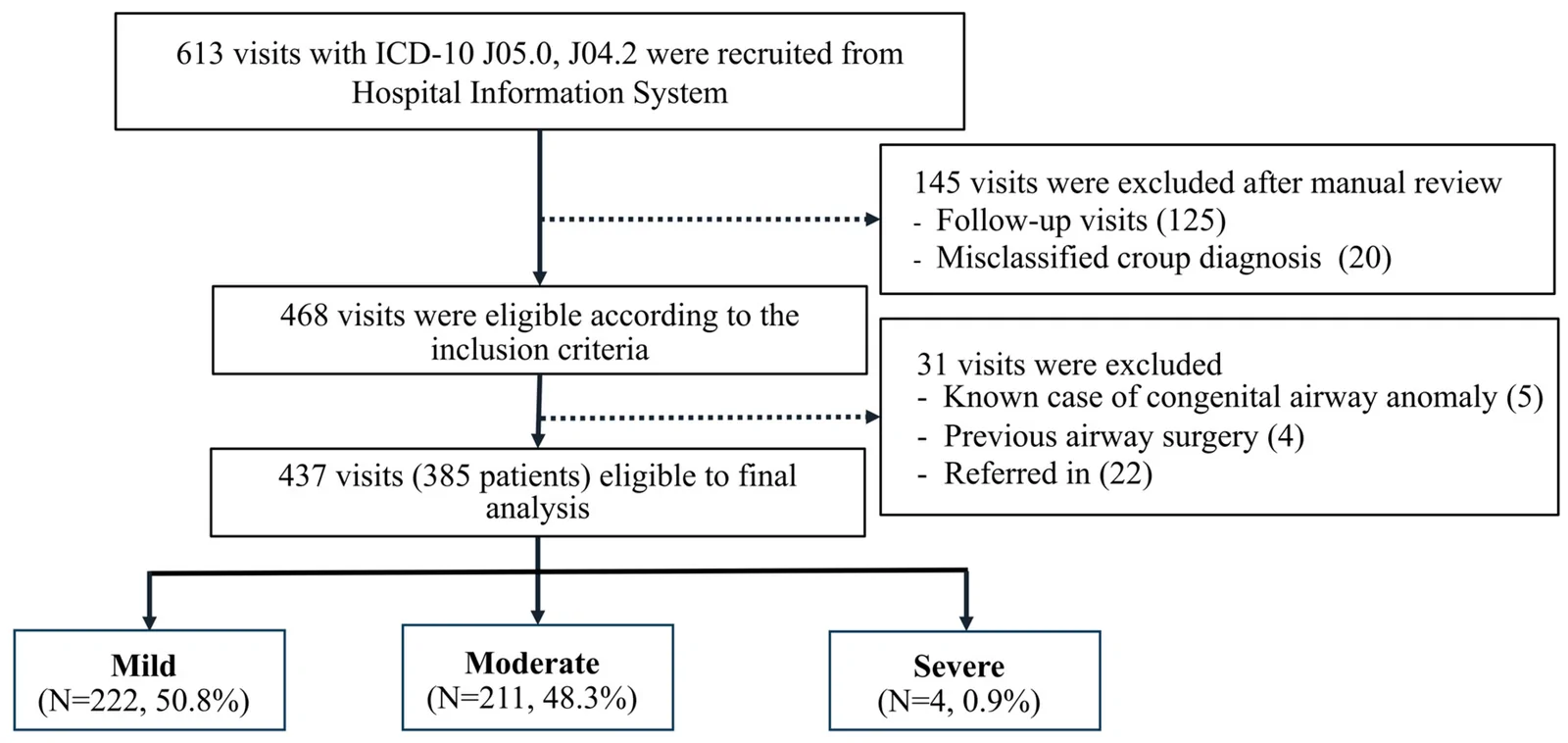
Flowchart of study participant selection.

**Figure 3:**
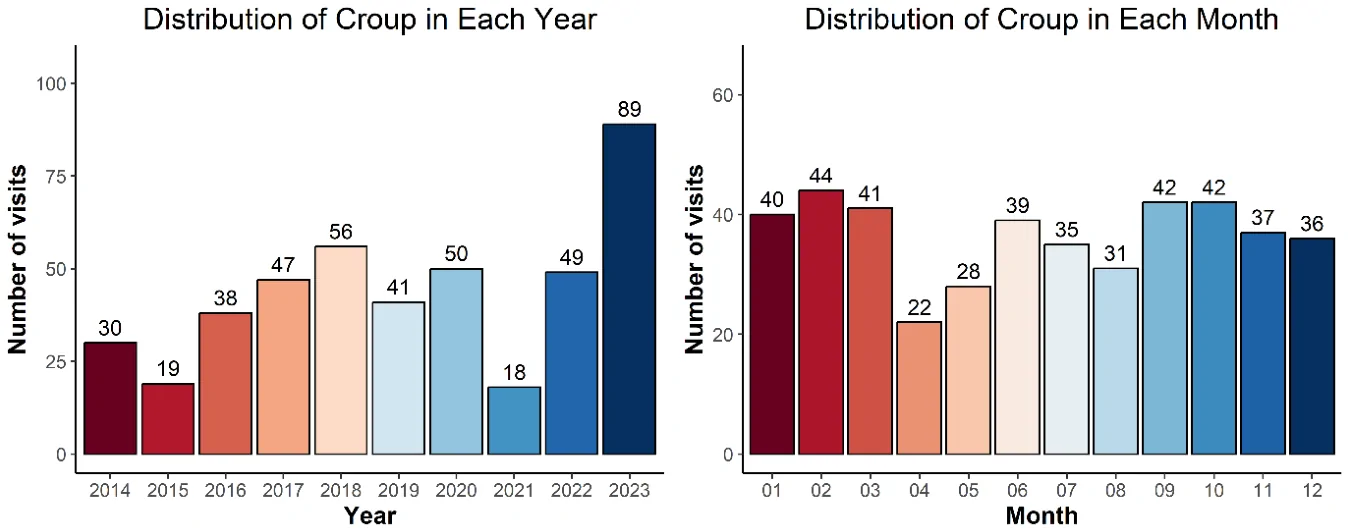
Distribution of croup diagnoses by year and month.

A total of 613 visits were initially identified through the HIS from 1 January 2014 to 31 December 2023. Following manual chart review, 437 visits by 385 patients were included in the final analysis (Figure 2). Of these, 222 (50.8%), 211 (48.3%), and 4 (0.9%) visits were classified into the mild, moderate, and severe croup categories, respectively. The lowest number of cases was noted between 2019 and 2021, with a marked increase in 2023. April and May had the lowest number of croup presentations (Figure 3).

### 3.1. Baseline Characteristics of Participants

Among the 437 visits, 311 (71.2%) involved male patients, with a mean age of 25.6 months (SD, 19). Most croup events (311/437, 71.2%) presented to the ED and were initially managed by general practitioners (GPs) or emergency physicians (EPs) (317/437, 72.5%). Corticosteroids and L-epinephrine NB were prescribed in 82.2% (359/437) and 64.5% (282/437) of visits, respectively. Oral dexamethasone was the most prescribed corticosteroid, followed by intravenous dexamethasone. Underdosing occurred in 59 of 359 (16.5%) corticosteroid prescriptions and 29 of 282 (10.3%) epinephrine NB prescriptions. Overdosing was observed in four (1.1%) corticosteroid cases and five (1.8%) epinephrine NB cases (Table S2). The overall hospital admission rate at the initial visit was 31.6%.

The treatment characteristics and clinical outcomes stratified by croup severity are presented in Table 1. Patients with moderate-to-severe croup were primarily managed in the ED, whereas those with mild croup were more frequently treated in the OPD. The use of croup-related medications and admission rates increased proportionally with disease severity. Bronchodilators and radiography were more commonly used in moderate croup, while antibiotics were prescribed more frequently in mild croup.

### 3.2. Adherence to National CPGs

**Figure 4:**
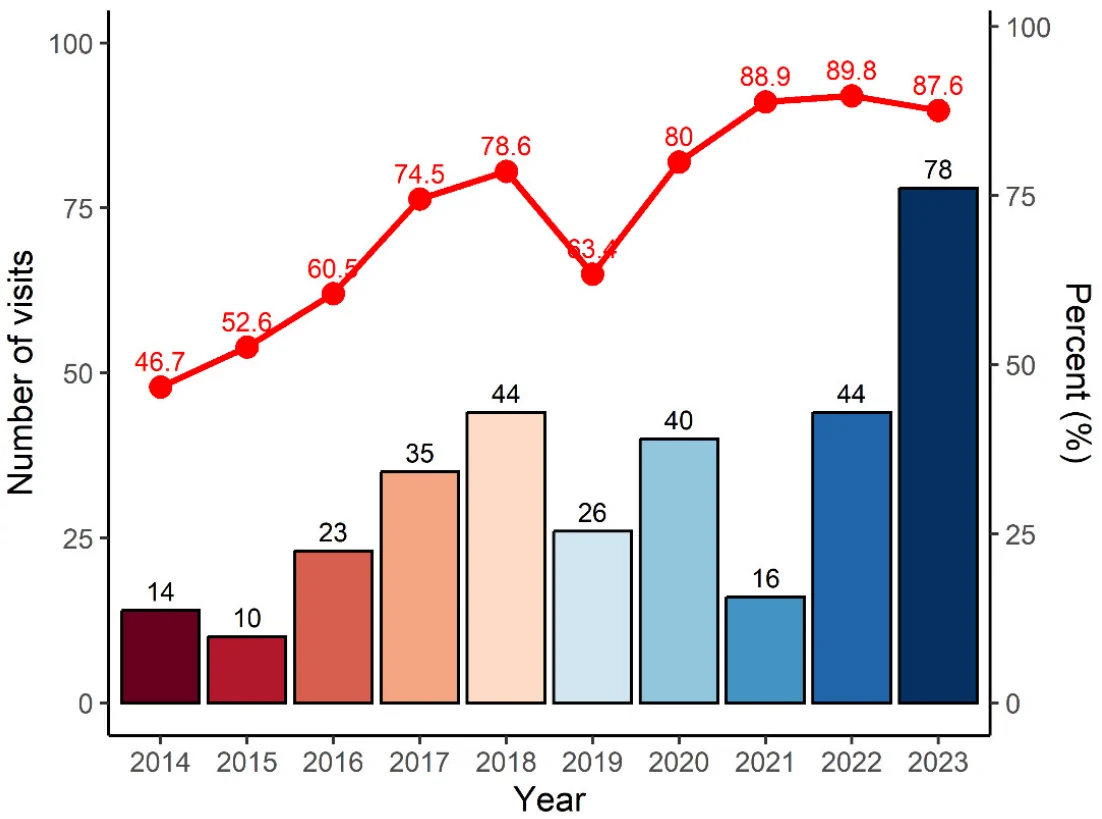
Frequency and percentages of complete adherence to the Thailand clinical practice guidelines for croup management.

Complete adherence to the national CPGs over the 10-year study period increased from 46.7% in 2014 to 87.6% in 2023 with 75.5% of overall adherence (Figure 4). Complete adherence rates were 69.8%, 81.0%, and 100% in the mild, moderate, and severe groups, respectively. Adherence to corticosteroid prescribing was lowest (359/437 visits, 82.2%) compared to those with epinephrine NB administration and observation time (99.1% and 92.9%, respectively). The mild group exhibited lower corticosteroid adherence than the moderate-to-severe group (69.8% [155/222 visits] vs. 94.4% [204/215 visits]). Conversely, observation time had the lowest adherence in the moderate group (85.6% [184/215 visits]) (Table S3). All treatments were completed in the severe group (100% [4/4 visits]).

Univariable analysis identified the risk factors for incomplete adherence: first episode of croup diagnosis (odds ratio [OR], 2.37; 95% confidence interval [CI], 1.15–5.54), paediatrician as the primary physician (OR, 2.61; 95% CI, 1.64–4.14), first visit to the OPD (OR, 2.36; 95% CI, 1.49–3.73), mild croup (OR, 1.89; 95% CI, 1.21–2.98), and year of visit (OR, 2.41; 95% CI, 1.55–3.78) (Table 2).

### 3.3. Risk Factors for Unplanned Revisits

Among the 299 patients discharged after the first visit, 59 (19.7%) had an unplanned revisit within 72 h, including 37 (16.7%) with mild croup and 22 (10.9%) with moderate croup. Of these 59 revisits, 28 visits (47.5%) occurred within 24 h, with either an increase in croup severity or no improvement, necessitating an additional dose of epinephrine nebulisation. Twenty-three visits (38.9%) were due to croup-related complications, including bacterial bronchitis, pneumonia, or diarrhoea. Eighteen patients (30.5%) were subsequently admitted during the second visit.

The risk factors for an unplanned revisit within 72 h were a GP as the primary physician (OR, 2.89; 95% CI, 1.45–6.3), first visit to the ED (OR, 3.38; 95% CI, 1.7–7.37), afternoon arrival time (OR, 3.23; 95% CI, 1.58–7.00), visit during 2014–2018 (OR, 1.63; 95% CI, 0.92–2.91), and receipt of repeated epinephrine NB during the first visit (OR, 2.56; 95% CI, 1.22–5.23). Croup severity, corticosteroid type and dose, and overall guideline adherence showed no significant association with unplanned revisits (Table 3).

## 4. Discussion

This study demonstrated a substantial improvement in adherence to the national CPGs for croup management, increasing from 46.7% in 2014 to 87.6% in 2023. Notably, adherence remained stable despite the post-COVID-19 rise in croup cases in 2023. Among the three adherence criteria, corticosteroid prescription showed the lowest adherence, particularly in the mild group. Factors associated with unplanned revisits included repeated doses of epinephrine during the first visit, treatment by GPs, initial visit to the ED, afternoon arrival time, and earlier years in the study period (2014–2018).

The improvement in adherence over the 10-year period was attributed to two factors. First, the 2019 transition to online CPG dissemination via the Paediatric Society of Thailand website increased accessibility. This was particularly helpful for GPs compared with the previously distributed handbook format. Second, the implementation of lectures on paediatric emergency management for physicians during their internship at our hospital strengthened their knowledge and confidence in the management of croup. This is consistent with previous research that shows provider knowledge as a key factor in CPG adherence [12]. Our adherence rates align with those of an Australian study that reported 65.9% and 91.1% adherence among GPs and EPs, respectively [11]. In contrast, a study from Italy reported much lower adherence rates (7.1%, 28.0%, and 40.9% in mild, moderate, and severe cases, respectively) [12]. However, that study included a variety of provider types in different settings where most providers were unfamiliar with croup management.

Although 82.2% of the patients in our study received corticosteroids, this treatment showed the lowest adherence, particularly in cases classified as mild. This finding aligns with the results from two studies conducted in Australia [11,13]. In those studies, adherence to corticosteroid prescription guidelines was notably lower in the mild group compared to patients with moderate-to-severe croup (45% vs. 97%) [13]. This could be due to the transitional change in practice from the previous national CPGs (before 2014), which did not recommend the use of corticosteroids for mild-severity croup. Moreover, mild croup was mainly treated by paediatricians in the OPD who are more familiar with children’s symptoms, leading to less awareness of corticosteroid prescriptions for croup treatment compared with EPs. This finding was consistent with two previous studies which also reported lower adherence regarding corticosteroids among paediatricians than among EPs [6,14]. Meanwhile, nebulised budesonide was rarely used in our study. This contrasts with findings from Italy, where inhaled corticosteroids were commonly used in mild-to-moderate croup (77–88%) [12]. The underuse of budesonide inhalers in Thailand may result from limited availability and lack of government coverage.

Nebulised epinephrine was the second most commonly administered treatment in this study. Although other international CPGs advise against its use in mild croup [11,12,13], 30% of mild cases in our study received epinephrine, a proportion significantly higher than the 2.3% reported in Italy [12]. Most of these prescriptions were from GPs, possibly due to discomfort caused by paediatric respiratory symptoms. Hoarseness accompanied by inspiratory stridor may heighten parents’ anxiety and lead clinicians to adopt a more aggressive approach to achieve rapid symptom relief.

While our protocol recommends 4 h of monitoring after epinephrine administration for patients with moderate-to-severe croup, 14.4% of cases did not meet the recommended observation period. This likely reflects the practical constraints of the ED workflow. Previous retrospective studies have demonstrated short observation times in the ED even for moderate cases [15,16,17]. One study reported a median time of 1 h 25 min (IQR, 47 min to 2 h 35 min) [15], and another reported a mean time of 2.8 h (SD, 1.8–4) [16]. In the U.S., 70% of CPGs recommend <2 h observation in the ED [6], aligning with the pharmacodynamics of epinephrine, which typically acts for 1–2 h [8]. Given the low incidence of rebound symptoms beyond this period, a shorter observation period may be appropriate for moderate croup and warrants further consideration in national CPG revisions.

Despite CPG recommendations against routine interventions, such as radiography, viral swab panels, or complete blood counts, and non-evidence-based medications (bronchodilators, antitussives, and antibiotics), inappropriate management was observed in 4–30% of the patients in this study. This corroborates the results of previous studies [6,11,18,19,20]. Prentice et al. [11] reported 33.7% radiography orders, 16.7% bronchodilator use, and 7.2% antibiotic prescriptions in Australia, while Tyler et al. [19] reported 21% neck radiography use in the U.S. Although CPGs and order sets were employed in the hospital guidelines, the rate of radiography could not be reduced [18]. In our study, the antibiotic prescriptions, predominantly macrolides, were more common among patients with mild croup who presented to the OPD and were evaluated by paediatricians. Most of these patients had clinical features suggestive of concomitant bacterial infection, including fever lasting more than 5 days, staccato cough, wheezing, or an injected tympanic membrane, which may have influenced the decision to prescribe antibiotics. A study from Korea [14] revealed higher antibiotic use by paediatricians (25.1%) than by EPs (3.13%), which was similar to our results. However, this finding contrasts with U.S. data demonstrating higher antibiotic use by EPs (13.0% vs. 8.6%) [6]. This highlights the need for ongoing evidence-based education to decrease unnecessary treatment and healthcare costs. Thus, emphasising this knowledge will be important for physicians in the future.

Unplanned revisits are another indicator of quality improvement in croup management. The rate of unplanned revisits was 19.7%, with 30% of patients requiring admission upon return. This rate is higher than the 5% revisit rate reported by Rosychuk et al. [15]. Our study’s definition of unplanned revisit differed from those used in previous studies. While earlier studies counted only unplanned emergency-department (ED) visits [15], we included all planned and unplanned revisits that required croup-related interventions. Nearly half of the unplanned revisits (28/59) occurred within 24 h of the initial discharge. Of these, 15 patients experienced persistent or worsening croup symptoms that necessitated an additional dose of nebulized epinephrine. The remaining patients presented with croup-related complications, such as diarrhoea, lower respiratory tract infections, or secondary bacterial infections. Risk factors included initial ED visits, management by GPs, especially during the afternoon shift, and repeated administration of epinephrine NB. However, the role of repeated epinephrine NB administration in determining admission remains debatable. Some guidelines recommend inpatient admission for children requiring > 2 doses of epinephrine [6]. A previous study found no significant difference in the revisit rate between patients receiving one or multiple doses of epinephrine [16,21]. Moreover, among asymptomatic patients admitted solely based on the number of epinephrine NBs, only 36% required a clinically important inpatient intervention, and 75% received only a single dose of epinephrine [16]. Another retrospective study found lower 7-day revisit rates in patients receiving multiple doses (0.8%) than in those who received a single dose (5.4%) [21]. These findings indicate that repeated epinephrine administration does not always indicate the need for admission and may be compatible with outpatient management. We found no association between short observation times and 72 h revisits, consistent with Udoh et al. [17] who reported no increased revisit risk with an observation time of <2 h in both mild and moderate cases. This further might support the reconsideration of observation duration in moderate croup in real-world practice.

One strength of this study is its large sample size. All details of croup, including severity, interventions, and treatment, were available in the HIS. However, our study had some limitations. First, it was conducted in a university-affiliated tertiary hospital, in which adherence rates may be higher than those in other settings due to academic oversight and specialist availability. National data would provide a clearer picture of adherence in croup management among different settings. Second, missing information such as medical history, family history, birth details, and history of recurrent croup may have influenced treatment outcomes. Third, only 64.5% of patients discharged after their initial visit attended the scheduled follow-up appointment within 72 h. Consequently, our findings may have underestimated the rate of unplanned revisits, particularly among patients who sought care at other hospitals. Last, the factors associated with incomplete adherence and unplanned revisits were identified based on univariable analyses. Although we performed multivariable analyses, only a limited number of variables could be included in the models because collinearity was detected among several predictors.

According to our findings, we propose the following recommendations for future guideline revisions. First, the dose of epinephrine NB should be standardised. We found that the rate of improper dosing of epinephrine NB was 12.1%, although it was not associated with unfavourable outcomes. A standard volume-based regimen according to age group (2.5 mL in patients aged < 4 years and 5 mL in patients aged ≥ 4 years) would be more practical than weight-based calculations. Second, further consideration of patient experience with repeated epinephrine NB is required. In hospitals with limited beds, early follow-up within 24 h is recommended.

This study highlights the overall improvements in croup management and adherence to national guidelines over the past decade. However, persistent suboptimal treatment practices and relatively high revisit rates underscore the need for targeted interventions to enhance guideline-based care, particularly among GPs and patients managed in emergency settings.

## Figures and Tables

**Table 1:** Baseline characteristics, treatment, and outcomes of participants (N = 437).

Variables	Total (N = 437)	Croup Severity		*p*-Value
		Mild (N = 222)	Moderate-to-Severe (N = 215)	
**Baseline characteristics**				
Age, months	25.6 ± 19	25 (12, 44.8)	19 (12, 36)	0.069
Male, *n* (%)	311 (71.2)	155 (69.8)	156 (72.6)	0.599
General practitioner as the primary physician, *n* (%)	317 (72.5)	132 (59.5)	185 (86.0)	<0.001
First visit at the emergency department, *n* (%)	311 (71.2)	123 (55.4)	188 (87.4)	<0.001
**Croup treatments**				
Corticosteroid, *n* (%)	359 (82.2)	155 (69.8)	204 (94.8)	<0.001
Improper dose of corticosteroid, *n* (%)	63 (17.6)	50 (32.7)	13 (6.0)	<0.001
Epinephrine, *n* (%)	282 (64.5)	71 (32.0)	211 (98.1)	<0.001
Improper dose of epinephrine, *n* (%)	34 (12.1)	9 (12.7)	25 (11.6)	0.471
Repeated L-epinephrine, *n* (%)	115 (26.3)	11 (5.0)	104 (48.3)	<0.001
Bronchodilator, *n* (%)	80 (18.3)	37 (16.7)	43 (20.0)	0.481
Antibiotic, *n* (%)	17 (3.9)	16 (7.2)	1 (0.5)	<0.001
Radiography, *n* (%)	143 (32.7)	47 (21.2)	96 (44.6)	<0.001
**Outcomes**				
Unplanned revisit, *n* (%)	59 (19.7)	37 (16.7)	22 (10.2)	0.203
Admitted, *n* (%)	138 (31.6)	13 (5.9)	125 (58.1)	<0.001

Continuous variables are presented as mean ± standard deviation or median (interquartile range), as appropriate.

**Table 2:** Risk factors for incomplete adherence during the first visit (N = 437).

Variables	Complete Adherence (N = 330)	Incomplete Adherence (N = 107)	*p*-Value
Age, months	22 (13, 42)	21 (10, 36)	0.096
Male, *n* (%)	236 (71.5)	75 (70.1)	0.873
History of croup, *n* (%)	53 (16.1)	8 (7.5)	0.039
Paediatrician as the primary physician, *n* (%)	74 (22.4)	43 (43)	<0.001
First visit at OPD, *n* (%)	80 (24.2)	46 (43)	<0.001
Mild severity, *n* (%)	155 (47)	67 (62.6)	0.013
Time of visit, *n* (%)			0.275
Morning shift (8.00–15.59)	107 (32.4)	43 (40.2)	
Afternoon shift (16.00–23.59)	129 (39.1)	34 (31.8)	
Night shift (24.00–7.59)	94 (28.5)	30 (28)	
Year of visit, *n* (%)			<0.001
2014–2018	126 (38.2)	64 (59.8)	
2019–2023	204 (61.8)	43 (40.2)	

Continuous variables are presented as mean ± standard deviation or median (interquartile range), as appropriate. OPD, outpatient department.

**Table 3:** Risk factors for unplanned revisits (N = 299).

Variables	Unplanned Revisit		*p*-Value
	No (N = 240)	Yes (N = 59)	
Age, months	24 (12, 44)	31 (16, 45)	0.296
Male, *n* (%)	174 (72.5)	41 (69.5)	0.765
General practitioner as the primary physician, *n* (%)	151 (62.9)	49 (83.1)	0.005
First visit at the ED, *n* (%)	142 (59.2)	49 (83.1)	0.001
Moderate severity, *n* (%)	68 (28.3)	22 (37.3)	0.236
Time of visit, *n* (%)			0.006
Morning shift (08.00–15.59)	95 (39.6)	12 (20.3)	
Afternoon shift (16.00–23.59)	71 (29.6)	29 (49.2)	
Night shift (24.00–07.59)	74 (30.8)	18 (30.5)	
Year of visit, *n* (%)			0.005
2014–2018	97 (40.4)	49 (83.1)	
2019–2023	143 (59.6)	10 (16.9)	
Corticosteroid, *n* (%)	190 (79.2)	44 (74.6)	0.555
No dexamethasone used, *n* (%)	113 (47.1)	29 (49.2)	0.889
Improper dose of corticosteroid, *n* (%) *	45 (23.8)	12 (27.9)	0.714
Improper dose of epinephrine, *n* (%) *	14 (12.2)	4 (10.5)	1.000
Repeat epinephrine nebulisation, *n* (%)	26 (10.8)	14 (23.7)	0.017
Observation time at the first visit, *n* (%)			0.332
<2 h	162 (67.5)	34 (57.6)	
2–4 h	62 (25.8)	19 (32.2)	
>4 h	16 (6.7)	6 (10.2)	
Bronchodilator used, *n* (%)	33 (13.8)	12 (20.3)	0.287
Antibiotic used, *n* (%)	15 (6.2)	1 (1.7)	0.211
Complete adherence, *n* (%)	170 (70.8)	36 (61.0)	0.193

* Improper doses of corticosteroids and epinephrine included both underdoses and overdoses. Continuous variables are presented as mean ± SD or median (interquartile range), as appropriate.

## Data Availability

The datasets used and/or analysed during the current study are available from the corresponding author on reasonable request. The data are not publicly available due to participant’s privacy.

## Supplementary Materials

The following supporting information can be downloaded at: https://www.mdpi.com/article/10.3390/children13081046/s1, Table S1: Diagnosis and procedures according to ICD-10-CM and ICD-9-CM for exclusion criteria; Table S2: Baseline Characteristics and Treatment of Participants (N = 437); Table S3: Adherence to Thai Clinical Practice Guideline of Croup (N = 437).

## Abbreviations

CPG	Clinical Practice Guideline
CI	Confidence Interval
ED	Emergency Department
EP	Emergency Physician
GP	General Practitioner
HIS	Hospital Information System
ICD-10-CM	International Classification of Diseases, 10th Revision, Clinical Modification
IQR	Interquartile Range
NB	Nebulisation
OR	Odds Ratio
OPD	Outpatient Department
SD	Standard Deviation
STROBE	Strengthening the Reporting of Observational Studies in Epidemiology
