# Supplementary material for: A Decade of Croup Management: Guideline Adherence, Practice Variation, and Opportunities for Improvement
Source: Children (Basel). 2026 Aug 6;13(8):1046. doi: 10.3390/children13081046 (PMC13511231; doi:10.3390/children13081046)
Supplement: Supplementary file 1 [file children-13-01046-s001.zip › children-4434149-supplementary.pdf]

## Supplement data

**Table S1.** Diagnosis and procedures according to ICD-10-CM and ICD-9-CM for exclusion criteria.

| **ICD-10** | **Description** |
|---|---|
| J38.0 | Paralysis of vocal cords and larynx |
| J38.2 | Nodules of vocal cord |
| J38.6 | Stenosis of larynx |
| Q30.0 | Choanal atresia |
| Q30.1 | Agenesis and underdevelopment of nose |
| Q30.2 | Fissured, notched and cleft nose |
| Q30.3 | Congenital perforated nasal septum |
| Q30.8 | Other congenital malformation of nose |
| Q30.9 | Congenital malformation of nose, unspecified |
| Q31.0 | Web of larynx |
| Q31.1 | Congenital subglottic stenosis |
| Q31.2 | Laryngeal hypoplasia |
| Q31.3 | Laryngocele |
| Q31.5 | Congenital laryngomalacia |
| Q31.8 | Other congenital malformation of larynx |
| Q31.9 | Congenital malformations of larynx, unspecified |
| Q32.0 | Congenital tracheomalacia |
| Q32.1 | Other congenital malformations of trachea |
| Q32.2 | Congenital bronchomalacia |
| Q32.3 | Congenital stenosis of bronchus |
| Q32.4 | Other congenital malformations of bronchus |
| Q39.1 | Atresia of oesophagus with trachea-oesophageal fistula |
| Q39.2 | Congenital trachea-oesophageal fistula without atresia |
| S11.0 | Open wound involving larynx and trachea |
| Z93.0 | Tracheostomy status |
| J95.5 | Postprocedural subglottic stenosis |
| J95.8 | Other postprocedural respiratory disorders |
| J95.81 | Postprocedural tracheal stenosis |
| J95.9 | Postprocedural respiratory disorders, unspecified |
| ICD-9 | |
| 31.1 | Temporary tracheostomy |
| 31.43 | Closed (endoscopic) biopsy of larynx |
| 31.72 | Closure of external fistula of trachea |
| 31.74 | Revision of tracheostomy |
| 31.92 | Lysis of adhesions of larynx or trachea |
| 31.98 | Dilatation of larynx |
| 97.23 | Replacement of tracheostomy tube |
| 97.37 | Removal of tracheostomy tube |
| 98.14 | Removal of foreign body in the larynx |
| 98.15 | Removal of foreign body in the trachea and bronchus |

**Table S2.** Baseline Characteristics and Treatment of Participants (N = 437).

| Characteristics | Values |
|---|---|
| Age, month | 25.6 (19) |
| Male, n (%) | 311 (71.2) |
| Previous history, n (%) | |
| Croup diagnosis | 61 (14) |
| Intubation | 24 (5.5) |
| Preterm infant | 17 (3.9) |
| First visit at emergency room, n (%) | 311 (71.2) |
| General physician as primary physician, n (%) | 317 (72.5) |
| X-ray prescription, n (%) | |
| None | 294 (67.3) |
| Neck | 4 (0.9) |
| Chest | 113 (25.9) |
| Both neck and chest | 26 (5.9) |
| Corticosteroid prescription, n (%) | 359 (82.2) |
| Type of corticosteroid, n (%) | |
| Prednisolone oral | 80 (18.3) |
| Dexamethasone oral | 113 (25.9) |
| Dexamethasone intramuscular | 61 (14) |
| Dexamethasone intravenous | 104 (23.8) |
| Budesonide nebulization | 1 (0.2) |
| Dose of corticosteroid, n (%) | |
| Under dose | 59 (16.5) |
| Proper dose | 294 (87.9) |
| Overdose | 4 (1.1) |
| Epinephrine nebulization, n (%) | 282 (64.5) |
| Dose of epinephrine nebulization, n (%) | |
| Under dose | 29 (10.3) |
| Proper dose | 248 (87.9) |
| Overdose | 5 (1.8) |
| Repeated epinephrine nebulization, n (%) | 115 (26.3) |
| Interval between the first and second dose of epinephrine, mins | 57.1 (37) |
| Time spends in emergency room/OPD, hours | 2.3 (2.2) |
| Bronchodilator nebulization, n (%) | 80 (18.3) |
| Antibiotic prescription, n (%) | 17 (3.9) |
| Appointment within 72 hour after discharge, n (%)* | 193 (64.5) |
| Unplanned revisit, n (%)* | 59 (19.7) |
| Admission, n (%) | 138 (31.6) |

* Calculated from 299 visits who were discharged at 1the first visit. Median with standard deviation was used for continuous variables

**Table S3.** Adherence to Thai Clinical Practice Guideline of Croup (N = 437).

| Criteria | Values |
|---|---|
| Corticosteroid in any severity, n (%) | 359 (82.2) |
| Epinephrine nebulization in moderate to severe group, n (%)* | 211 (98.1) |
| Observed time more than 4 hours in moderate to severe group, n (%)* | 184 (85.6) |

* Calculated from 215 visits with moderate to severe croup.
